# Sentence completion in progressive supranuclear palsy following transcranial direct current stimulation

**DOI:** 10.1038/s41531-023-00610-0

**Published:** 2023-12-09

**Authors:** Johanna Scholtz, Sabine Weiss, Christoph Redecker, Horst M. Müller

**Affiliations:** 1 Experimental Neurolinguistics Group, Bielefeld University, Bielefeld, Germany; 2 Clinical Linguistics, Bielefeld University, Bielefeld, Germany; 3 Department of Neurology, Hospital Lippe-Lemgo, Lemgo, Germany

**Keywords:** Movement disorders, Rehabilitation, Translational research

## Abstract

Progressive supranuclear palsy (PSP) is an atypical Parkinsonian disorder which results in deterioration of motor and cognitive skills, including language disorders such as impaired word retrieval. While there is evidence of successful use of tDCS to improve word fluency in PSP, little is known about the effectiveness of brain stimulation for word retrieval in sentence context. Therefore, we investigated whether tDCS reduces sentence completion time in PSP patients. In this sham-controlled, triple-blinded crossover study, anodal tDCS (atDCS) was applied over the left Broca’s area at 2 mA for 20 min *(n* = 23). In contrast to patients with multiple system atrophy (MSA), also an atypical Parkinsonian disorder, and healthy elderlies, sentence completion improved in PSP patients when tDCS was applied. The improvement in word fluency reported in previous studies using other electrode positions was not replicated. By using atDCS of the left Broca’s area, we were able to demonstrate a difference between the two movement disorders. The obtained insight could be helpful to improve language therapy of these disorders.

## Introduction

Atypical Parkinsonian disorders share common features of Parkinson’s disease, as they can cause bradykinesia, rigidity, resting tremor, and postural instability^1^. In addition, these diseases are specified by more variable symptoms that lead to a diagnosis of either Lewy body dementia, corticobasal degeneration, multiple system atrophy (MSA), or progressive supranuclear palsy (PSP).

PSP is classified as a neurodegenerative disorder with tau proteinopathy^1^, that primarily damages the basal ganglia, but also areas of the frontal, parietal, and temporal lobes, as well as parts of the brainstem and cerebellum^2^. Characteristic symptoms of PSP include oculomotor dysfunction, akinesia, and postural instability. Besides that, cognitive dysfunction is another important symptom of PSP which includes abnormalities of frontal function as well as language disorders^1,3^. The clinical presentation of PSP can vary, which is why different subtypes of PSP have been introduced to classify the clinical characteristics^1^. While some subtypes are characterized more by different motor symptoms, others show more limitations in cognitive and language abilities.

Executive dysfunction is the most common cognitive impairment in PSP^4^ and may occur early in the disease^5^. Of 52 PSP patients examined by Ou et al.^6^, 76,9% displayed executive deficits. The extent of these executive disorders correlates with the severity of the disease. Similarly, the degree of language impairment in PSP varies from almost no impairment and severe language disorder.

Some patients show reduced output on simple sentence structures that may develop into agrammatism^6,7^. Compared to control subjects, PSP patients generally use fewer words but pronouns more frequently^8^. In addition, PSP can lead to impaired sentence completion and difficulties in sentence generation^9,10^. Comprehension deficits at the sentence level have also been described by several authors^11,12^, as well as naming errors^7^. Semantic impairments are of particular interest in the study of language impairment in PSP, as they can be detected even in otherwise preclinical patients^8^. At the pragmatic level, narrative language may be impaired^9^ making conversational tasks crucial for diagnosing language disorders in PSP^7^. Interestingly, some patients with PSP can develop deficits at the discourse level when generating connected sentences without being impaired at the word or sentence level^13,14^. These deficits might be explained by the concept of energization^14^. In contrast to patients with other neurodegenerative diseases and healthy elderlies, some PSP patients have difficulty maintaining the required high level of attention when performing a language task over a longer period of time^15^. This influences performance on tasks demanding the production of multiple connected sentences, such as the description of a picture. Another language deficit in PSP that has been frequently investigated is reduced word fluency^7^, typically assessed with phonemic and semantic fluency tasks. Both task types show decreased fluency, although there is ongoing debate as to which of the task types is more impaired in PSP^4,9–12^. Impaired energization might also explain reduced these deficits in PSP^15^.

For example, in multiple system atrophy (MSA), another atypical Parkinsonian disease, the limitations in fluency are mostly due to deficits in phonemic fluency^4^. In the present study, patients with MSA were included as control subjects. MSA is an α-synucleinopathy that mainly affects subcortical brain areas such as the putamen, pons, and cerebellum^16^. Cortical atrophies are not considered a classic feature of MSA, although atrophies of the frontal and temporal lobes have been described by Jellinger^17^. In earlier stages of the disease, cognitive deficits are less common in MSA than in other neurodegenerative disorders^18^, but do exist nevertheless, as shown by Brown et al.^5^. Of 398 patients with MSA, 31,8% showed cognitive impairments such as deficits in attentional and executive tasks. According to Lazzeri et al.^19^, dementia is present in up to 15% of MSA patients, generally manifesting in the later disease stages. Besides word fluency, language disorders affecting naming, repetition, and reading as well as simple sentence structure have been described in MSA^20^.

Unfortunately, little is known to date about therapeutic approaches to improve speech and language impairment in patients with atypical Parkinsonian disorders. One approach to support classical speech and language therapy is the additional application of transcranial direct current stimulation (tDCS), a non-invasive method of electrical brain stimulation. A direct current flows through electrodes on the scalp, that alters the resting membrane potential of neurons near the electrodes^21^. TDCS can be used as either excitatory (anodal tDCS; atDCS) or inhibitory (cathodal tDCS; ctDCS) stimulation. In atDCS, the resting membrane potential of stimulated neurons tends to be depolarized, which may improve performance on tasks executed during stimulation^22^. In ctDCS, the resting membrane potential is hyperpolarized, reducing cortical excitability. TDCS is a very safe method with comparably minor side effects^23^. The method is increasingly used for investigating language impairment and its treatment in patients with neurodegenerative disorders (for a review^24^); [^25^; Heimann et al., presented at 12th World Congress for Neurorehabilitation, 2022].

Recently, tDCS has also been used for improving language skills in patients with PSP. Alexoudi et al.^26^ applied atDCS to motor and premotor cortex and described improvement in both phonemic and semantic fluency. Madden et al.^27^ applied atDCS to the left dorsolateral prefrontal cortex (DLPFC) and reported improvement in phonemic fluency and action naming. Valero-Cabré et al.^28^ investigated the effect of both excitatory and ctDCS on the DLPFC. They demonstrated that atDCS on the left hemisphere improved phonemic fluency, whereas ctDCS on the right hemisphere boosted semantic category judgement. These studies mainly investigated the influence of tDCS on word fluency retrieving single items. However, these tasks do not take into account language reception and sentence production, as well as more complex executive processes.

In the current study, we therefore decided to extend the word retrieval experiments to include sentence completion tasks. In these tasks, patients have to insert the searched item into a complete sentence. To do this, they must analyze both the semantic context and the syntactic structure of the sentences presented to them in order to choose a semantically and syntactically appropriate sentence ending. Thus, both language reception and the ability to build well-formed sentences are necessary for successfully performing a sentence completion task. In addition, executive functions such as attention and working memory are also required, which, as mentioned earlier, may be impaired in these patients.

We investigated the effect of tDCS on sentence completion in patients with PSP. In doing so, we hypothesized that atDCS over the left Broca’s area would lead to a reduction in sentence completion time. The left Broca’s area is involved in word selection^29^ as well as syntactic working memory^30^ and control of word production^31^, all processes important for sentence completion. We expected that under the influence of tDCS, patients would find a suitable sentence ending faster than during sham stimulation.

To compare our results with those of previous studies of tDCS in PSP, we included word fluency as another word retrieval task. Here, we expected that our results would show the same improvement in phonemic and/or semantic word fluency as in the previously described studies. Our participants should be able to retrieve more words under the influence of tDCS than under sham stimulation.

Because we hypothesized a relationship between cognitive deficits in PSP and the efficacy of tDCS, we assumed that tDCS would not improve word retrieval in persons with little or no cognitive deficits according to the literature. To test this hypothesis, we decided to compare the receptivity of patients with PSP to tDCS with the receptivity of patients with a different disorder from the same disease group. For this, we included patients with MSA in our study since MSA, like PSP, is an atypical Parkinsonian disorder but usually does not lead to cognitive deficits in earlier stages of the disease. To exclude the possibility of a general stimulation effect independent of health status, we also included age-matched healthy subjects. Here, we expected that healthy elderly participants would benefit little or not at all from tDCS when performing rather simple word retrieval tasks.

## Results

### Demographic and clinical characteristics

Five patients with PSP were recruited for this study (Supplementary Table 1). To compare the effect of tDCS on sentence completion, four patients with MSA and 14 healthy elderlies (HE) also participated as control subjects (Table 1).

**Table 1:** Demographic data and results of the diagnostic tests (mean ± SD) of patients with PSP MSA and healthy elderlies.

	PSP	MSA	HE
Number of participants	5	4	14
Sex: f/m	1/4	3/1	9/5
Mean age (in years)	72.6 ± 5.8	55.8 ± 4.1	64.5 ± 13.3
Mean duration of illness (in years)	3.6 ± 1.7	4.5 ± 1.7	−
MoCA (cut-off value 26)	18.8 ± 5.8	26.5 ± 2.7	26.2 ± 1.7
KOPS (max. 240)	190 ± 29.9	234 ± 2.8	236 ± 1.7
Spontaneous speech (content words per phrase)	1.3 ± 0.5	2.0 ± 0.2	2.0 ± 0.4
Spontaneous speech (word count)	170.0 ± 128.9	259.8 ± 18.5	280.8 ± 25.1
Spontaneous speech (type-token-ratio)	0.5 ± 0.02	0.6 ± 0.04	0.5 ± 0.3
Spontaneous speech (time for 50 phrases in s)	370.2 ± 175.4	271.8 ± 28.3	143.9 ± 28.8
Semantic fluency	9.6 ± 4.6	23.0 ± 3.5	25.5 ± 6.3
Phonemic fluency	4.3 ± 2.7	12.6 ± 2.3	18.1 ± 3.4
Phonemic fluency with category shift	8.8 ± 6.0	18.0 ± 4.8	22.1 ± 5.1

*PSP* Progressive supranuclear palsy, *MSA* Multiple system atrophy, *HE* healthy elderlies, *MoCA* Montreal Cognitive Assessment, *KOPS* Communicative-pragmatic screening for patients with aphasia (Kommunikativ-pragmatisches Screening für Patienten mit Aphasie).

There was a significant age difference between PSP and MSA patients (*t* = 4.87, *p* = 0.002). This was because the time of onset of the disease was earlier in MSA. Comparing the other groups, no significant age difference was found. Regarding sex, there was also no significant difference between the three groups. Furthermore, there was no overall difference in illness duration between PSP and MSA.

### Diagnostics of speech, language and cognitive functions

In the MoCA^32^, PSP patients reached a significantly lower score than patients with MSA as well as HE. No significant difference was found between MSA and HE.

The KOPS^33^ is a German screening tool for examining verbal, nonverbal, and compensatory skills and was used to depict possible pragmatic impairments of our subjects. The communication skills of the PSP group were significantly decreased in comparison to the MSA group and the HE group. In contrast, the communication skills of patients with MSA and HE did not differ significantly.

The ratio of content words per phrase was significantly lower for PSP in contrast to MSA and HE. No significant difference was found between MSA and HE. The word count within 50 phrases of spontaneous speech did not differ comparing MSA to PSP or HE. Yet, the PSP group produced a significantly lower number of words than the HE group. The type-token ratio was significantly lower for PSP in contrast to MSA as well as HE, but no significant difference was detected for MSA and HE. The HE group was significantly faster in uttering the 50 analyzed phrases than the PSP and MSA group. No significant difference in articulation time was found for PSP and MSA.

Moreover, we used the RWT^34^, a German assessment for word fluency and tested both phonemic and semantic fluency, and phonemic category shift. Semantic word fluency showed a significantly lower number of responses for PSP in comparison to MSA and HE, respectively. No significant difference was found between MSA and HE. For phonemic word fluency, we also identified significantly less responses in the PSP group compared to the MSA and HE group. In contrast to the semantic task, the lexical task also displayed reduced word fluency for the MSA group compared to the HE group. A test of phonemic fluency with category shift did not indicate a significant difference between MSA and HE, but we recorded significantly less responses for PSP in contrast to MSA as well as HE. Statistical comparisons are illustrated in Table 2.

**Table 2:** Statistical comparison between groups for the diagnostic tasks.

Groups per task	*t*	*p*
*MoCA*		
PSP vs. MSA	−3.63	**0.002**
PSP vs. HE	16.78	**<0.001**
MSA vs. HE	−0.19	0.850
*KOPS*		
PSP vs. MSA	2.87	**0.010**
PSP vs. HE	3.78	**0.001**
MSA vs. HE	0.11	0.914
*Spontaneous speech (content words per phrase)*		
PSP vs. MSA	2.96	**0.008**
PSP vs. HE	3.79	**0.001**
MSA vs. HE	−0.02	0.987
*Spontaneous speech (word count)*		
PSP vs. MSA	1.36	0.215
PSP vs. HE	−3.21	**0.005**
MSA vs. HE	1.54	0.142
*Spontaneous speech (type-token-ratio)*		
PSP vs. MSA	2.69	**0.031**
PSP vs. HE	−3.70	**0.002**
MSA vs. HE	−0.04	0.969
*Spontaneous speech (time for 50 phrases in s)*		
PSP vs. MSA	1.10	0.309
PSP vs. HE	4.89	**<0.001**
MSA vs. HE	7.84	**<0.001**
*Phonemic fluency*		
PSP vs. MSA	4.95	**0.002**
PSP vs. HE	−8.19	**<0.001**
MSA vs. HE	3.03	**0.008**
*Semantic fluency*		
PSP vs. MSA	−4.76	**0.002**
PSP vs. HE	−5.08	**<0.001**
MSA vs. HE	0.47	0.473
*Phonemic switching*		
PSP vs. MSA	2.48	**0.042**
PSP vs. HE	− 4.84	**<0.001**
MSA vs. HE	1.46	0.164

*PSP* Progressive supranuclear palsy, *MSA* Multiple system atrophy, *HE* healthy elderlies, *MoCA* Montreal Cognitive Assessment, *KOPS* Communicative-pragmatic screening for patients with aphasia (Kommunikativ-pragmatisches Screening für Patienten mit Aphasie).

### Effect of stimulation on experimental tasks

The generalized linear model revealed no overall stimulation effect for the sentence completion task across all three participant groups (*X²* = 2.23*, df* = 1, *p* = 0.135). However, a significant group effect (*X²* = 242.74, *df* = 2, *p* < 0.001) and a significant stimulation x group interaction (*X²* = 13.98, *df* = 2, *p* < 0.001) were found. The covariate age was significant (*X²* = 42.54, *df* = 1, *p* < 0.001).

The PSP group completed sentences significantly slower than the HE group. Moreover, the MSA group was significantly slower than the HE group in this regard. No significant difference was found between PSP and MSA patients in overall sentence completion (Table 4).

**Fig. 1:**
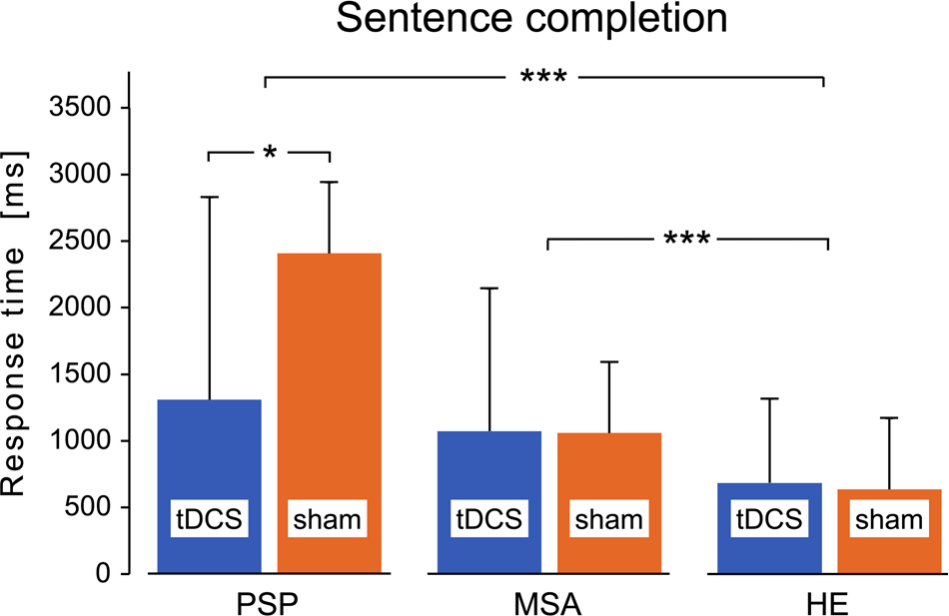
Mean response time during sentence completion in the PSP, MSA, and HE groups. In MSA and HE, no difference was found between tDCS and sham stimulation. In the PSP group, sentence completion time was significantly shorter when tDCS was applied. *PSP* Progressive supranuclear palsy, *MSA* Multiple system atrophy, *HE* healthy elderlies. Data are results of generalized linear models with log-link function and gamma distribution. *p < 0.05, **p < 0.01, ***p < 0.001. The error bars represent standard deviation.

When tDCS was applied, PSP patients were significantly faster at sentence completion compared to sham stimulation (*z* = 3.039, *p* = 0.017). The other two groups did not show differences in sentence completion during tDCS compared to sham (Fig. 1).

Analysis of the individual data of the PSP patients showed tDCS was a significant predictor of faster sentence completion in some patients. Therefore, we wanted to determine whether there was a relationship between disease duration (since diagnosis) and the effectivity of tDCS in the sentence completion task in PSP patients (Table 3). To do this, we performed a partial correlation analysis with age as a possible confounding variable. A significant correlation (Pearsons’s *r* = 0.99, *p* = 0.011) was found between disease duration (in years) and the probability of a significant difference (z-value) between sentence completion during tDCS compared to sham.

**Table 3:** Duration of illness since diagnosis mean sentence completion time and results of generalized linear models in individual PSP patients during sham and tDCS.

DOI (in years)	sham	tDCS	GLM
2	780	901	*B* = −0.018; *z* = −0.529; *p* = 0.598
2	1454	976	*B* = 0.008; *z* = 0.302; *p* = 0.763
4	1238	601	*B* = 0.066; *z* = 3.23; ***p*** = **0.002**
4	5664	2753	*B* = 0.062; *z* = 1.99; ***p*** = **0.047**
6	5063	2421	*B* = 0.089; *z* = 4.91; ***p*** < **0.001**

*DOI* duration of Illness, *GLM* generalized linear model. Sentence completion time is measured in ms.

P values in bold are significant after generalized linear modelling with log-link function and gamma distribution.

For the motor control task (verbal reaction time), there was neither a significant stimulation effect nor a stimulation x group interaction. The significant group effect (*X²* = 1018.05, *df* = 2, *p* < 0.001) was due to the fact that the PSP patients were significantly slower than the MSA and HE groups. In addition, the MSA group was significantly slower to respond to the stimuli than the HE group. The covariate age was not a significant predictor of verbal reaction time.

Concerning the three word fluency tasks (RWT), neither a general effect of stimulation nor an interaction between group and stimulation was found. Also, the covariate age was no predictor of general word fluency. However, a group effect was found for phonemic and semantic word fluency as well as for the phonemic switching tasks. On all three tasks, the PSP group retrieved significantly fewer items than the MSA and HE groups. The MSA group retrieved significantly fewer items than the HE only on phonematic WF. Statistical comparisons are illustrated in Table 4.

**Table 4:** Statistical comparison between groups for the experimental tasks.

Groups per task	*z*	*p*
*Sentence completion*		
PSP vs. MSA	1.13	0.260
PSP vs. HE	12.40	**<0.001**
MSA vs. HE	10.66	**<0.001**
*Verbal reaction time*		
PSP vs. MSA	8.21	**<0.001**
PSP vs. HE	29.22	**<0.001**
MSA vs. HE	13.56	**<0.001**
*Phonemic fluency*		
PSP vs. MSA	3.01	**0.001**
PSP vs. HE	7.91	**<0.001**
MSA vs. HE	3.72	**0.001**
*Semantic fluency*		
PSP vs. MSA	3.56	**0.002**
PSP vs. HE	6.26	**<0.001**
MSA vs. HE	1.55	0.373
*Phonemic switching*		
PSP vs. MSA	3.90	**0.001**
PSP vs. HE	7.48	**<0.001**
MSA vs. HE	2.27	0.087

*PSP* Progressive supranuclear palsy, *MSA* Multiple system atrophy, *HE* healthy elderlies.

## Discussion

Our results showed that patients with PSP had significantly shorter sentence completion times when tDCS was applied. This result could be used in speech and language therapy as an extension of the ability to compensate for word retrieval difficulties. Because naming errors are a language symptom of PSP^7^, patients with PSP are at high risk of being temporarily unable to complete their own sentences due to impaired word retrieval. They could therefore benefit from practicing sentence completion and the use of tDCS could potentially enhance this practice effect.

According to the results of our diagnostics (Table 1, Supplementary Table 1), the PSP group in this study displays many of the language impairments typical of this disorder. The low scores in the MoCA^32^, with 4 out 5 patients below the cut-off value for cognitive impairment, demonstrate executive dysfunction in PSP^4–6^. The results in the KOPS^33^ show why conversational tasks should be included in PSP diagnostics^7^, given the performance of our PSP group on these communicative-pragmatic tasks. With the evaluation of spontaneous speech samples, we were able to provide evidence for discourse deficits in connected sentences in PSP^13,14^. Lastly, the results of our word fluency tasks conform with reports on impaired fluency in PSP^7^, with lower performance on phonemic than on semantic fluency^4,11,12^. The results of our diagnostics, especially on spontaneous speech and fluency, could be linked to an impaired energization process when generating verbal responses over a long period of time^14,15^.

Due to the speech, language, and cognitive deficits of the PSP patients discussed above, differences between them and the control groups are found in the experimental tasks. Group effects were found for the motor control task, the fluency tasks, and sentence completion. In general, the PSP group was significantly slower in the motor control task and produced fewer items in the fluency tasks than the HE and the MSA groups. Motor reaction time was slower in the PSP than in the MSA and again slower in the MSA than in the HE group. Since no influence of age was found here, these results could be possibly due to the general lower processing speed and motor abilities of the different groups due to neurodegeneration of frontal cortical and subcortical regions^2,4,16,18^.

However, in sentence completion, the PSP group was significantly slower than the HE group but interestingly not than the MSA group. The MSA group was also slower than the HE group. For the group effect on sentence completion, we identified age as a significant predictor, meaning that participants with older age were slower overall than younger participants, regardless of which group they belonged to. However, there was no significant age difference between PSP and HE. If age were the only predictor of performance on sentence completion, the reported significant difference in sentence completion time between PSP and HE would not exist. Also, that the younger MSA group was significantly slower than the older HE group cannot be explained by the age effect alone. Therefore, the age effect should not be considered as the only factor influencing performance in sentence completion, but rather the neurodegenerative disease of PSP and MSA patients and the concomitant cognitive-linguistic deterioration. This assumption is supported by the fact that the age effect was not significant in the motor control and fluency tasks. Here, the group effects presented could be influenced by the nature of the disorders and their impairments in both cognitive and language abilities. Therefore, a combined effect of age and disease can be hypothesized for slow sentence completion in PSP.

Under the influence of tDCS, PSP patients were significantly faster on sentence completion than under sham stimulation. The MSA patients showed no improvement with tDCS, although both the PSP and MSA groups performed significantly slower than the HE group on the sentence completion task.

In the PSP group the verbal reaction time in the motor control task was not reduced by tDCS. Therefore, it can be assumed that the improvement reported for sentence completion was not simply due to improved verbal reaction time. This promotes the idea that the improvement of sentence completion was due to improved word retrieval, among other factors. It is reasonable to assume that other cognitive functions that affect sentence completion could have also been enhanced by tDCS. Besides word retrieval, sentence completion also relies on cognitive abilities like verbal working memory, the syntactic-semantic analysis of presented items, and word selection combined with lexical inhibition. As the left Broca’s area is associated with syntactic working memory^30^, control processes of word production^31^, and word selection^29^, the application of tDCS might have had a facilitatory effect on these cognitive tasks, resulting in improved sentence completion.

Improved energization might have also been responsible for the results. As described by Barker et al.^15^, maintaining the high level of attention needed to generate language is impaired in PSP. With a highly demanding task like sentence completion, it is not surprising that PSP patients performed significantly worse than the HE group, given that this task also requires a high level of attention repeatedly. Due to energization being associated with the frontal lobe^15^, tDCS of this brain area might have had a stimulatory effect on the level of energization, which could have positively influenced sentence completion.

Interestingly, tDCS had no influence on sentence completion time in patients with MSA and in healthy participants. This difference could be explained by the choice of stimulation site and the brain areas damaged differently in PSP and MSA. As mentioned above, we chose the left Broca’s area as the stimulation site because the function of predominantly frontal and temporal lobes is affected in PSP patients^2^. In contrast to the frequently described cortical damage in PSP, cortical degeneration is not considered a predominant characteristic of MSA^18^, although diffuse cortical atrophy is sometimes described^17^. Therefore, it could be that fronto-cortical stimulation in MSA patients does not have the same effect as in PSP patients with lesions in the stimulated area. Stimulation of the left Broca’s area appears to be relevant for sentence completion in patients with PSP.

Additional performance improvement with tDCS is more likely with weaker baseline performance^35^. This could be the reason why no improvement in sentence completion time was found with tDCS in the healthy elderlies, since they had a better baseline. Yet, even though the MSA group also had a weaker baseline than HE group, improvement was only detected in the PSP group. This reinforces the theory of the importance of the stimulation site chosen. In this respect, it can be assumed that a slow baseline in sentence completion is not solely accountable for whether tDCS improved sentence completion.

Another reason for the different response to tDCS could be the fact that the PSP and MSA groups differed regarding their mean age. In the PSP group, the mean age was 73 years, whereas the mean age in the MSA group was 56 years. Furthermore, the MSA patients also performed better on our cognition and language tests due to their comparatively younger age. While the MSA group was still in an age range where language skills remain comparatively constant, the older PSP group was already in an age range where these skills are decreasing^36^. However, the fact that no influence of tDCS was found in HE argues against the sole influence of age on the success of stimulation. In conclusion, the covariate age influenced the participants’ performance on sentence completion in general. However, it did not influence whether sentence completion was additionally improved by tDCS or not.

To investigate whether all PSP patients respond equally to tDCS, we analyzed the individual data and found different responsiveness of patients. The literature discusses why some individuals respond very well to tDCS and some less well^37^. Several explanations can be found. Since age had no influence on the individual responsiveness of our PSP patients based on our analyses, we suspected that it could be the duration of disease, which might correlate with the severity of the disease. The PSP patients in our study had a disease duration of two to six years, and the longer the disease duration, the more pronounced the effect of tDCS on sentence completion time.

In contrast to earlier studies, tDCS did not improve phonemic or semantic word fluency or phonematic switching in our PSP patients. This was also true for the MSA patients and the healthy subjects. The effectiveness of tDCS on word fluency in patients with PSP has been previously shown^26–28^. One explanation for the difference between our results and these previous studies could be the stimulation of different brain regions. Madden et al.^27^ and Valero-Cabré et al.^28^ used the left dorsolateral prefrontal cortex (DLPFC) and both groups reported enhanced phonemic word fluency. An obvious explanation would be that the DLPFC is more important for phonemic fluency than the left Broca’s region. However, other studies also reported an increase in phonemic and partly also semantic word fluency by atDCS of left inferior frontal regions [Heimann et al., presented at 12th World Congress for Neurorehabilitation; ^38^];. Another explanation for the lack of fluency enhancement in our study could be the frequency of stimulation. While in our study subjects were treated with tDCS only once, Madden et al.^27^ reported a single case study with two days of tDCS, while Alexoudi et al.^26^ applied tDCS for 10 consecutive days. Yet, Valero-Cabré et al.^28^ also stimulated with tDCS for one session and reported enhancement of phonemic fluency. Alexoudi et al.^26^ stimulated the motor and premotor cortex and reported an excitatory effect on both phonemic and semantic fluency. Due to the function of the motor and premotor cortex, and because Alexoudi et al.^26^ did not evaluate the effect of tDCS on verbal reaction time, the described effect on both semantic and phonemic fluency might be due to reduced verbal reaction time rather than improved fluency itself.

Although the previously cited studies examined cognitive tasks other than word fluency, no group controlled for verbal reaction time. Valero-Cabré et al.^28^ conducted a control task in which participants were asked to generate visual sequences by selecting items on a tactile screen. In their experiment, no significant influence of tDCS was found on this task. Although reaction time might have influenced performance in the group’s control task, it is more likely that the task measures general motor response than verbal response, because the task requires finger movements and not speech. Madden et al.^27^ aimed to rule out the possibility that language improvements were influenced by improved motor production. For this, they used a reading task and measured the words uttered per minute. The authors did not find significant changes in reading while applying tDCS. While this might be closer to the execution of word fluency tasks than the approach of Valero-Cabré et al.^28^, the focus on motor speed of word production does not, in our view, capture the cognitive aspect of accelerated speed in word retrieval. In their experiment, Alexoudi et al.^26^ found significant improvements in several other cognitive tasks in addition to significant improvements in letter fluency. This suggests that the application of tDCS in their experiment affected not only letter fluency specifically, but also general cognitive abilities, visuo-motor activity, processing speed, auditory verbal memory, and learning. Especially, processing speed and general cognitive abilities might influence performance on letter fluency tasks, so the discussed improvement of letter fluency might also be due to other improved mental abilities.

In summary, to our understanding, the previous studies that investigated word fluency in PSP under the influence of tDCS did not control for the important aspect of verbal reaction time. Therefore, it may be possible that the improvement in word fluency was enhanced by decreased verbal reaction time.

In addition, the general language skills of the patients in our study differed from those of the subjects in the earlier studies. Since our PSP group was rather heterogenous in disease duration, cognitive level, and language skills (Supplementary Table 1), the mean performance of our PSP patients on language tasks was lower than that of subjects in the previous studies. Because we were not able to replicate the improvements in fluency reported earlier^26–28^, it could be hypothesized that sentence completion improves most at lower baseline performance levels, whereas fluency tends to improve when subjects perform at higher baseline performance levels. Thus, the effect of tDCS on word fluency in patients with PSP remains an unanswered question. In the future, other studies addressing this issue should differentiate the duration of language tasks and compare the stimulation frequency and subjects’ overall language skills more systematically.

Despite the promising results regarding the increase of sentence completion time in PSP patients, some limitations of the study must be considered. First, due to the low incidence of atypical Parkinsonian disorders, our sample size of five patients with PSP and four patients with MSA was small. Second, due to the heterogeneity of language impairments in PSP^8^, it was hard to obtain a homogenous group of study participants. Third, we used a study design with only one day of tDCS application whereas the efficacy of the stimulation could certainly be increased within multiple days of stimulation.

Furthermore, the MSA patients were younger than the other two other groups and age was found to influence sentence completion time. The first symptoms of PSP normally occur at the mean age of 63 years^39^, whereas MSA usually begins at an earlier age^17^. Yet, median survival time is not very different and is approximately 7 years for PSP^39^ and 6 to 10 years for MSA^17^. Therefore, it is not surprising that comparatively older PSP and younger MSA patients were recruited during data acquisition for this study.

Because we lacked data on standardized PSP rating scales, we could only assess disease severity by subjective diagnosis of speech, language and cognitive data. Therefore, we also included disease duration in our analyses which sometimes may not correspond to disease severity. Moreover, we also did not have data concerning the PSP subtype for all subjects. Therefore, correlations between subtype and language deficits and between subtype and efficacy of tDCS cannot be established. It can be assumed that our PSP subjects with low performance on language tasks might be classified as PSP-SL, which highlights speech and language dysfunction. Based on their performance on the MoCA^32^, their disorder could also be classified as the PSP-F subtype with a focus on cognitive and behavioral deficits. Thus, the PSP subjects with a better performance in our language tasks could be assigned to one of the other subtypes of PSP. Further studies need to analyze the efficiency of tDCS considering PSP subtypes.

Another aspect to be considered in future studies is the inclusion of other subject groups with slow sentence completion (e.g., MCI) for testing whether the effect of tDCS on sentence completion is specific to PSP or also applies to other diseases.

Processes underlying sentence completion were enhanced in PSP patients by atDCS over the left Broca’s area. This suggests that, particularly in PSP, anodal stimulation of the left inferior frontal gyrus accelerates sentence completion in contrast to other word retrieval tasks such as word fluency. To the best of our knowledge, this paper is the first to examine the efficiency of tDCS on sentence completion in PSP, controlling for age and motor reaction time, for example. Despite the small sample, our results are of clinical relevance because the effect of tDCS on sentence completion in PSP is very promising. These results can be used to extend language and speech therapy by combining conventional language exercises and tDCS to improve patients’ compensation skills and thus to slow the decline in language abilities due to neurodegenerative disease.

## Methods

### Study participants

This study included 5 patients with PSP, 4 patients with MSA, and 14 healthy elderlies (Table 1). The diagnosis of PSP or MSA was made by experienced neurologists according to international diagnostic criteria^1^. There are no data on the diagnosis of specific PSP and MSA subtypes. Also, we did not obtain information on the severity of the disorders according to standardized scales. For this reason, we focused on the diagnosis of speech, language, and communication in the patients. The participants were informed about the tDCS study both orally and in writing and provided their written consent for participation and usage of data. Written consent was obtained for the publication of patient photographs. The study was approved by the Ethics Committee of Bielefeld University, Germany (approval number 2021-072) and was conducted according to the Declaration of Helsinki.

### Diagnostic and experimental tasks

Our study included three appointments over a three-week period (Fig. 3). At the first appointment, our subjects underwent several tests (Table 1), which lasted approximately 90 min and included the German version of the MoCA^32^ to control for severe cognitive deficits as well as the evaluation of communication-pragmatic skills with the KOPS^33^. In addition, word fluency was evaluated using the RWT^34^.

Furthermore, we collected data on spontaneous speech among all three groups and analyzed the first 50 phrases of a semi-standardized interview about the participants’ experiences during the COVID-19 pandemic. This topic was chosen because it was universal and it can be assumed that each respondent had certain experiences during this period. We assessed the ratio of content words per phrase, expecting that patients used fewer words combined with a higher number of pronouns. Other measures included the word count in 50 phrases, the type-token ratio, and the time taken to produce the analyzed 50 phrases.

In the experimental sessions, we used different items of the RWT^34^ to evaluate word fluency. For testing word retrieval in sentence context, we generated and pilot-tested a sentence completion task, as described below. To ensure that decreased response time in the sentence completion task was not due to a reduced voice onset time, a control verbal reaction time task was performed in both sessions. 15 audio signals with a frequency of 300 Hz and a duration of 200 ms were played through headphones in a temporally randomized manner. Participants had to answer “ja” (yes) as quickly as possible when they heard the signal. The time between the end of the audio signal and the onset of the response was analyzed.

### Sentence completion task

For the sentence completion task, 100 sentences were constructed with the last word missing (Supplementary Table 2, Supplementary Table 3). These were pseudorandomized and divided into two sets of 50 sentences each, with each set being assigned to either session 1 or 2. The two sets were matched using qualitative, instrumental, objective, and predicative relations, as well as meronomy and taxonomic relationships. Examples for each relationship type are given in Supplementary Table 2 and Supplementary Table 3. No significant difference was found in the distribution of sentence types between the two sessions (*χ*² = 0.338, *p* = 0.997). The words to be completed were nouns, verbs and adjectives/adverbs, with no significant difference between the sessions (*χ²* = 0.093, *p* = 0.955). Similarly, no significant differences were found for the number of words (*t* = −0.117, *p* = 0.908) and syllables in the sentences (*t* = 0.205, *p* = 0.838) or for the duration of articulation (*t* = 0.107, *p* = 0.915). A pilot study was conducted with 20 healthy participants (age Ø 37.5 ± 21.94) to determine the constraint of the sentences with respect to the words completed by them. The frequency of the determined endings was used to classify the constraint of the stimuli and to equalize it between sets. If the percentage of the most predominant sentence ending was less than 40%, the sentence was classified as a low constraint item. If the percentage was greater than 80%, the sentence was classified as a high constraint item. Any percentage in between was classified as medium constraint. The distribution of low, medium, and high constraint sentences did not differ significantly between the two sessions (*χ²* = 0.059, *p* = 0.97). The sentences were spoken in a soundproofed recording booth by a German native speaker (44.1 kHz/16 bit) and edited with Audacity software (version 2.4.1).

### Transcranial direct current stimulation

**Fig. 2:**
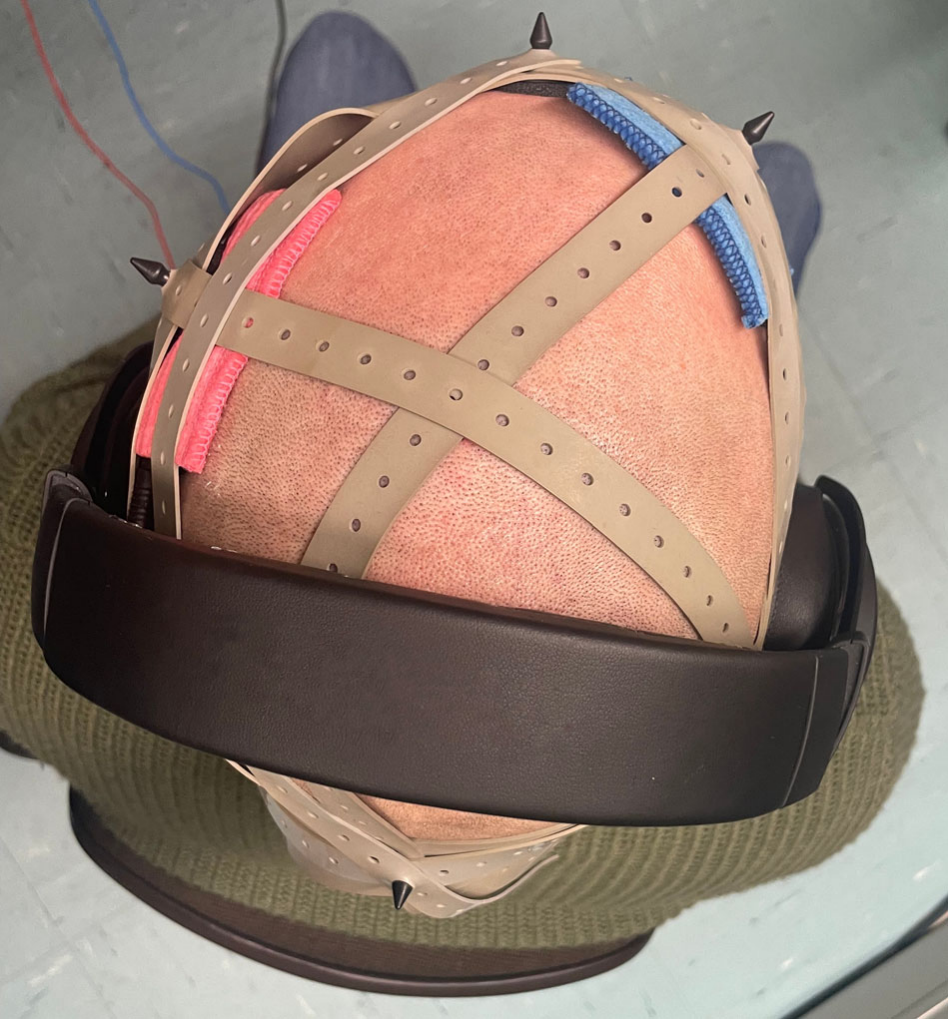
Placement of electrodes and headphones. Anode (red) over the left Broca region, cathode (blue) supraorbital on the right hemisphere. The headphones were placed by the experimenter, ensuring that they did not interfere with the placement of the electrodes or cables. Written consent for publication of this photograph was obtained.

TDCS was applied according to the international guidelines of Antal et al.^40^ and performed online during task performance using a battery-powered direct current stimulator (NeuroConn DC-Stimulator plus) with two electrodes (5 ×7 cm^2^) in sponges soaked with sodium chloride (0.9 %). The anode was placed with the long side horizontally over F3, F7, and T3 of the 10–20 EEG system to optimally cover Broca’s area^41^, and the cathode supraorbitally on the right hemisphere (FP2), also with the long side horizontally (Fig. 2). Direct current was applied at an intensity of 2 mA for a duration of 20 min with a fade-in and fade-out of 10 s. In the sham condition, the current started but was automatically ramped down after 30 s. Before and after stimulation, participants completed a questionnaire to assess possible side effects (e.g., itching, tingling, headache).

### Experimental procedure

**Fig. 3:**
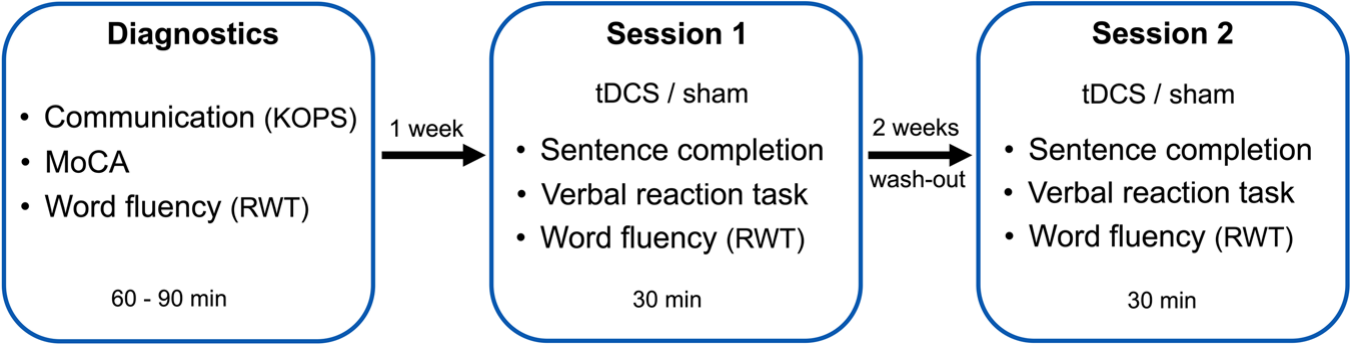
Study procedure. On the first appointment, the subjects underwent some tests for diagnostic purposes (KOPS, MoCA and RWT). One week later, they participated in session 1, in which sentence completion and verbal reaction time were tested in addition to word fluency, either with tDCS or sham stimulation. After a wash-out-time of two weeks, session 2 was conducted in the same way. The tasks were performed in randomized order. *KOPS* Communicative-pragmatic screening for patients with aphasia (Kommunikativ-pragmatisches Screening für Patienten mit Aphasie), MoCA Montreal Cognitive Assessment, RWT Regensburg word fluency test (Regensburger Wortflüssigkeitstest).

In this triple-blinded, sham-controlled crossover design, each participant completed three sessions (Fig. 3). After a diagnostic session, tDCS was applied in one session, and in the other session sham stimulation was used. There was a wash-out phase of two weeks between the two sessions to avoid a carry-over effect. The experiment took place either in the hospital Lippe-Lemgo, during a home visit, or in the laboratory of the experimental neurolinguistics group at Bielefeld University.

The two experimental sessions lasted approximately 60 min, including electrode application, and comprised two language tasks (sentence completion and word fluency using the RWT^27^), and a verbal reaction time task. In the sentence completion task, subjects were played sentences with missing final words over headphones and were asked to complete them verbally as quickly as possible. There was no time limit for completing the sentences to avoid frustration when word-finding difficulties occurred. Both the sentences played and the participants’ responses were recorded with a digital recorder (Olympus WS-852) and later analyzed manually using a sound editor (Audacity, ver. 2.4.1). The response time was considered to be the time between the ending of the item played and the beginning of the participant’s response.

### Statistical analysis

Data of 5 PSP patients, 4 MSA patients, and 14 healthy controls were included in the statistical analysis performed with Jamovi (Version 2.0.0.0; 2021). Sentence completion times were corrected for outliers (exclusion of data, above or below twice the standard deviation) and log-transformed. To determine whether participants showed increased sentence completion time during tDCS compared with sham stimulation, a generalized linear model with log-link function and gamma distribution was used. This procedure was also chosen for the influence of tDCS on verbal reaction time and word fluency tasks and was applied to the three groups. The variable age was included in all analyses as a covariate to control for its influence. The generalized linear models were used because the dependent variables (sentence completion time, verbal RT) in the group of healthy elderly participants and PSP patients had distributions that deviated significantly from the normal distribution (Shapiro-Wilk-tests). The log-link function was chosen because it allowed the AIC criterion of the models to be kept lower. The data from both the PSP- and MSA groups had positive scale values that were skewed toward larger positive values. Therefore, the gamma distribution was used. Statistical results are Bonferroni-corrected *p*-values.

### Supplementary information

Supplementary Material

Related Manuscript File

## Data Availability

The datasets used and/or analyzed during the current study are available from the corresponding author on reasonable request.
