# Supplementary material for: Sentence completion in progressive supranuclear palsy following transcranial direct current stimulation
Source: NPJ Parkinsons Dis. 2023 Dec 9;9:162. doi: 10.1038/s41531-023-00610-0 (PMC10710437; doi:10.1038/s41531-023-00610-0)
Supplement: Supplementary file 1 — Supplementary Material [file 41531_2023_610_MOESM1_ESM.pdf]

Supplementary Table 1. Results of the diagnostic tests for individual PSP patients.

	PSP1	*PSP2*	*PSP3*	*PSP4*	*PSP5*
MoCA (cut-off value 26)	19	27	17	11	20
KOPS (max. 240)	224	226	130	141	229
Spontaneous speech (content words per phrase)	1.46	1.82	0.66	0.88	1.56
Spontaneous speech (word count)	235	315	31	36	233
Spontaneous speech (type-token-ratio)	0.49	0.47	0.5	0.48	0.53
Spontaneous speech (time for 50 phrases in s)	175	302	370	653	351
Semantic fluency (mean for 1 & 2 minute task)	15.5	12.5	4.5	5.5	10
Phonemic fluency (mean for 1 & 2 minute task)	7	6.5	1	2	5
Phonemic fluency with category shift (2 minute task)	9	17	3	3	12

PSP Progressive Supranuclear Palsy, *MoCA* Montreal Cognitive Assessment, *KOPS* Communicative-pragmatic screening for patients with aphasia (Kommunikativ-pragmatisches Screening für Patienten mit Aphasie).

Supplementary Table 2. Set of stimuli for the sentence completion task, session 1.

Item	Most possible ending in pilot study (Percentage of most possible ending)	Sentence type	Constraint rated in pilot study	Frequency of most possible ending (according to the German dictionary)	Number of words in item (German)	Number of syllables in item (German)	Duration of articulation in item (German) (in ms)	Part of speech to be added
Der Hund des Nachbarn bellt besonders…<br><br>*The neighbor's dog barks particularly…*	laut<br>*loudly*<br>(75%)	qualitative relation	medium	5/7	7	9	226,3	adjective / adverb
Er beobachtet das Wild mit dem…<br><br>*He observes the wild game with the…*	Fernglas<br>*binoculars*<br>(90%)	instrumental relation	high	3/7	7	9	204,3	noun
Der Lehrling soll die Ware ausladen und…<br><br>*The apprentice is supposed to unload and … the goods*	(ein)sortieren<br>*sort*<br>(30%)	objective relation	low	4/7	8	11	256,6	verb
Der Bagger hat eine große…<br><br>*The excavator has a big…*	Schaufel<br>*shovel*<br>(95%)	meronymy	high	3/7	6	8	154,4	noun
Die Freundin schreibt den Brief mit dem…<br><br>*The girlfriend writes the letter with the…*	Füller<br>*fountain pen*<br>(60)	instrumental relation	medium	3/7	8	8	211,6	noun
Eine echtgoldene Uhr ist sehr…<br><br>*A gold watch is very…*	teuer / wertvoll<br>*expensive / valuable*<br>(35% each)	predicative relation	low	5/7	6	9	204,9	adjective / adverb
Die Wohnung hat einen sonnigen…<br><br>*The apartment has a sunny…*	Balkon<br>*balcony*<br>(50%)	meronymy	medium	4/7	6	9	186,9	noun
Der Weg zum Gipfel ist sehr…<br><br>*The way to the summit is very…*	steil<br>*steep*<br>(40%)	predicative relation	low	4/7	7	7	175,6	adjective / adverb
Die Jugendlichen beginnen ihre Ausbildung sehr…<br><br>*The teenagers start their apprenticeship very…*	früh<br>*early*<br>(40%)	qualitative relation	low	6/7	7	14	275,1	adjective / adverb
Der junge Mann gesteht der Dame seine…<br><br>*The young man confesses his… to the lady*	Liebe<br>*love*<br>(80%)	objective relation	high	5/7	8	11	218,1	noun

Der alte Richter gilt als recht…<br><br>*The old judge is regarded as quite…*	streng<br>*strict*<br>(60%)	qualitative relation	medium	5/7	7	8	206,1	adjective / adverb
*Obwohl sie ihm alles genau erklärte, hatte er dafür kein…<br><br>*Although she explained everything to him precisely, he did not have… for it*	Verständnis<br>*understanding*<br>(73%)	objective relation	-	5/7	11	17	334,4	noun
Der Hürdenläufer kontrolliert die Zeit mit der…<br><br>*The hurdler controlled the time with the…*	Stoppuhr<br>*stopwatch*<br>(90%)	instrumental relation	high	3/7	8	12	256,2	noun
*Der Manager hatte es eilig und schaute auf die…<br><br>*The manager was in a hurry and looked at the…*	Uhr<br>*watch or clock*<br>(100%)	objective relation	-	5/7	10	14	288,8	noun
Der Gast isst die Suppe mit dem…<br><br>*The guest ate the soup with the…*	Löffel<br>*spoon*<br>(90%)	instrumental relation	high	4/7	8	8	193	noun
Papageien sind sehr farbenprächtige…<br><br>*Parrots are very colorful…*	Vögel<br>*birds*<br>(85%)	taxonomy	high	5/7	5	11	232,7	noun
Die Mutter möchte gerne…<br><br>*The mother would like to…*	kochen / essen / schlafen<br>*cook / eat / sleep*<br>(10% each)	predicative relation	low	5/7	5	7	131	verb
Der Benzinpreis steigt ziemlich…<br><br>*The gas price increases rather…*	stark / schnell<br>*strongly / rapidly*<br>(15% each)	qualitative relation	low	6/7	5	7	209,3	adjective / adverb
Irland ist eine grüne …<br><br>*Ireland is a green…*	Insel<br>*island*<br>(85%)	taxonomy	high	5/7	5	7	162,8	noun
Der Metzger schneidet das Fleisch mit dem…<br><br>*The butcher cuts the meat with the…*	Messer<br>*knife*<br>(95%)	instrumental relation	high	4/7	8	9	225,2	noun
Der Koch würzt die Suppe recht…<br><br>*Then cook seasons the soup rather…*	stark<br>*strongly*<br>(35%)	qualitative relation	low	6/7	7	7	184	adjective / adverb

*Mit ihrer plötzlichen Kündigung setzte sie ein deutliches…<br><br>*With her sudden notice of termination, she set a clear…*	Zeichen<br>*signal*<br>(86%)	objective relation	-	5/7	9	16	308,2	noun
Der Patient hält das neue Medikament für…<br><br>*The patient believes the medicine to be…*	gut<br>*good*<br>(20%)	qualitative relation	low	6/7	8	12	246,7	adjective / adverb
Autoreifen sind aus…<br><br>*Car tires are made of…*	Gummi<br>*rubber*<br>(90%)	predicative relation	high	3/7	4	6	166,9	noun
Der Bauer erntet….<br><br>*The farmer is harvesting…*	Kartoffeln / die Kartoffeln / Gemüse<br>*potatoes / the potatoes / vegetables*<br>(15% each)	objective relation	low	4/7	4	5	134,1	noun
Im Wald kann man…<br><br>*In the forest, you can…*	spazieren<br>*stroll*<br>(25%)	predicative relation	low	4/7	5	4	175,7	verb
Seine Miene zeigte einen angespannten…<br><br>*His expression showed a tense…*	Ausdruck<br>*expression*<br>(55%)	meronymy	medium	5/7	6	12	233,9	noun
Der Kunde bezahlt die Rechnung mit…<br><br>*The customer paid the bill with...*	der Karte<br>*the card*<br>(20%)	instrumental relation	low	5/7	8	9	206,9	noun
Er liebt es, unterwegs im Auto Radio zu…<br><br>*He loves…to the radio in the car*	hören<br>*to listen*<br>(100%)	objective relation	high	5/7	9	10	294,8	verb
Die Kinder waschen sich die Hände mit…<br><br>*The children wash their hands with…*	Seife<br>*soap*<br>(75%)	instrumental relation	medium	3/7	8	10	200,6	noun
Eine Sommerwiese blüht…<br><br>*A summer meadow blooms…*	bunt<br>*colorfully*<br>(30%)	qualitative relation	low	5/7	4	7	166,8	adjective / adverb
*Der Kellner stellte die Blumen in eine…<br><br>*The waiter put the flowers in a…*	Vase<br>*vase*<br>(100%)	objective relation	-	3/7	8	11	219,1	noun

Im Kühlschrank sind noch Eier und…<br><br>*In the fridge, there are still eggs and…*	Milch<br>*milk*<br>(50%)	objective relation	medium	4/7	7	8	212,8	noun
Scharlach war früher eine besonders gefährliche …<br><br>*Scarlet fever used to be an especially dangerous...*	Krankheit<br>*illness*<br>(70%)	taxonomy	medium	5/7	7	14	279,5	noun
Er muss das hartgekochte Ei noch…<br><br>*He still has to … the hard boiled egg*	pellen<br>*peel*<br>(60%)	objective relation	medium	2/7	7	9	187,5	verb
Eine Mauer ist aus …<br><br>*A wall is made of…*	Stein<br>*stone*<br>(35%)	predicative relation	low	5/7	5	6	128,8	noun
Sie möchte dieses Jahr keine Marmelade…<br><br>*This year, she doesn't want to … jam*	kochen<br>*cook*<br>(40%)	objective relation	low	4/7	7	12	232,1	verb
Die Wendeltreppe hat ein…<br><br>*The spiral staircase has a…*	Geländer<br>*railing*<br>(60%)	meronymy	medium	3/7	5	7	156,7	noun
Das Mädchen will der Freundin etwas ins Ohr…<br><br>*The girl wants to … something in the friend's ear*	flüstern<br>*whisper*<br>(95%)	objective relation	high	4/7	9	11	250,8	verb
Der Koch schlägt die Buttercreme mit dem…<br><br>*The cook whips the butter cream with the…*	Schneebesen<br>*whisk*<br>(50%)	instrumental relation	medium	2/7	8	9	231,6	noun
Eine tiefe Wunde heilt meistens…<br><br>*A deep wound usually heals…*	langsam<br>*slowly*<br>(50%)	qualitative relation	medium	5/7	6	9	242,2	adjective / adverb
Die Mutter füttert…<br><br>*The mother is feeding…*	das Kind / das Baby<br>*the child / the baby*<br>(30% each)	objective relation	low	6/7	5	5	144,5	noun
*Wegen der Panne wechselte der Fahrer den…<br><br>*Due to the breakdown the driver changed the…*	Reifen<br>*tire*<br>(57%)	objective relation	-	4/7	8	11	242,6	noun

Der Zahnarzt entfernt den Karies mit dem…<br><br>*The dentist removes the cavity with the…*	Bohrer<br>*drill*<br>(75%)	instrumental relation	medium	3/7	8	11	267	noun
Im Urlaub wollen viele Leute…<br><br>*On vacation, many people like to…*	entspannen<br>*relax*<br>(40%)	predicative relation	low	4/7	6	9	191,4	verb
Der Gärtner gießt die Pflanzen mit…<br><br>*The gardener is watering the plants with…*	Wasser<br>*water*<br>(55%)	instrumental relation	medium	5/7	7	8	212,2	noun
Das Wochenende war warm und…<br><br>*The weekend was warm and…*	sonnig<br>*sunny*<br>(75%)	predicative relation	medium	4/7	6	8	178,3	adjective / adverb
Sie will den Kindern ein paar Brote…<br><br>*She wants to … make the kids a sandwich*	schmieren<br>*make*<br>(75%)	objective relation	medium	4/7	8	9	195,2	verb
Mein Nachbar wäscht sein Auto besonders…<br><br>*My neighbor washes his car particularly…*	gründlich<br>*thoroughly*<br>(45%)	qualitative relation	low	4/7	7	10	224,6	adjective / adverb
Gestern mussten wir…<br><br>*Yesterday we had to…*	wandern / Hausaufgaben machen / einkaufen / arbeiten / zuhause bleiben / lernen<br>*hike / do homework / go shopping / work/ stay at home / study*<br>(10% each)	predicative relation	low	5/7	4	5	116,9	verb

* These sentences were not tested in the pilot study. They were later added to the stimuli set to equalize the level of constraint for both sessions. To ensure that this table contains enough information about the constraint of the sentences, we added results from our experiment. The information on the frequency of the most probable word then refers to the result from our experiment. It has to be considered that the marked items include data acquired from patients with language disorders and therefore may not fully match the healthy group of the pilot study.

Supplementary Table 3. Set of stimuli for the sentence completion task, session 2.

Item	Most possible ending in pilot study (Percentage of most possible ending)	Sentence type	Constraint rated in pilot study	Frequency of most possible ending (according to the German dictionary)	Number of words in item (German)	Number of syllables in item (German)	Duration of articulation in item (German) (in ms)	Part of speech to be added
Die Froschmänner tauchen ziemlich…<br><br>*The male frogs dive rather…*	tief<br>*deeply*<br>(60%)	qualitative relation	medium	5/7	5	8	191,2	adjective / adverb
Die Kinder freuen sich auf die…<br><br>*The children are looking forward to…*	Ferien<br>*holidays*<br>(60%)	objective relation	medium	4/7	7	8	186,3	noun
Eine Flasche ist aus…<br><br>*A bottle is made of…*	Glas<br>*glass*<br>(90%)	predicative relation	low	4/7	5	6	141,6	noun
Die Mutter süßt die Erdbeeren mit…<br><br>*The mother sweetens the strawberries with…*	Zucker<br>*sugar*<br>(95%)	instrumental relation	high	4/7	7	9	196,2	noun
Der Förster fällt den Baum mit der…<br><br>*The ranger cuts the tree with the…*	Axt<br>*axe*<br>(45%)	instrumental relation	medium	3/7	8	8	211	noun
Sie will heute mit dem Bus zur Arbeit…<br><br>*Today she wants to go to work by…the bus*	fahren<br>*taking*<br>(100%)	objective relation	high	5/7	9	10	206,1	verb
Gewitterregen ist besonders…<br><br>*Thundershower is particularly…*	gemütlich / beruhigend, schön / heftig / stark / laut<br>*cozy / calming / nice / violent / strong / loud*<br>(10% each)	predicative relation	low	4/7	4	9	184,2	adjective / adverb
Die Kinder wollen unbedingt ein Eis…<br><br>*The children necessarily want to… ice cream*	essen<br>*eat*<br>(70%)	objective relation	medium	5/7	7	10	233,3	verb
Eine Autobahn ist eine besonders breite…<br><br>*A freeway is a particularly wide…*	Straße<br>*street*<br>(85%)	taxonomy	high	5/7	7	13	264,1	noun
Eine Kerze ist aus…<br><br>*A candle is made of…*	Wachs<br>*wax*<br>(90%)	predicative relation	high	3/7	5	6	143,8	noun
*Der Kranke bestand nur noch aus Haut und…	Knochen<br>*bones*<br>(96%)	predicative relation	-	4/7	9	10	244,9	noun

The sick person only consisted of skin and…								
Der Mechaniker kann…<br><br>*A mechanic can…*	reparieren<br>*repair*<br>(20%)	predicative relation	low	4/7	4	6	137,3	verb
Deine Stimme klingt am Telefon oft…<br><br>*On the telephone, your voice often sounds…*	anders<br>*different*<br>(30%)	qualitative relation	low	5/7	7	10	222,9	adjective / adverb
*Bei ihrer ersten Vorstellung vermittelte sie einen guten…<br><br>*At her first presentation, she gave a good…*	Eindruck<br>*impression*<br>(95%)	objective relation	-	5/7	9	17	312,3	noun
Die Schneiderin näht mit der…<br><br>*The seamstress sews with the…*	Nähmaschine<br>*sewing machine*<br>(90%)	instrumental relation	high	3/7	6	7	163,4	noun
Die alte Kirchturmuhr ist ziemlich…<br><br>*The old church clock is rather…*	laut<br>*loud*<br>(45%)	predicative relation	medium	5/7	6	9	216,8	adjective / adverb
*Obwohl er die Aussage mehrmals veränderte, ergab sie keinen…<br><br>*Although he changed the statement serveral times, it didn't make…*	Sinn<br>*sense*<br>(100%)	objective relation	-	5/7	10	18	364,6	noun
Der Bauer muss heute unbedingt…<br><br>*Today the farmer necessarily has to…*	ernten<br>*harvest*<br>(20%)	predicative relation	low	4/7	6	9	197,1	verb
Sie erreichen den zehnten Stock mit dem…<br><br>*They reach the tenth floor with the…*	Fahrstuhl<br>*elevator*<br>(75%)	instrumental relation	medium	4/7	8	10	216,8	noun
Hornissen haben einen langen…<br><br>*Hornets have a long…*	Stachel<br>*sting*<br>(95%)	meronymy	high	3/7	5	9	158,1	noun
Zum Kuchen will sie gleich die Sahne…<br><br>*For the cake, she wants to … the cream in a moment*	schlagen<br>*whip*<br>(50%)	objective relation	medium	5/7	8	9	211,6	verb
Die Leiter hat sehr viele…<br><br>*The ladder has lots of…*	Sprossen<br>*rungs*<br>(70%)	meronymy	medium	3/7	6	7	243,2	noun
Die Mädchen pflücken…	Blumen<br>*flowers*<br>(75%)	objective relation	medium	4/7	4	5	117,8	noun

The girls are picking…								
Im Herbst ist es oft kalt und….<br><br>*In autumn, it often is cold and…*	nass<br>*wet*<br>(35%)	predicative relation	low	4/7	8	7	201,6	adjective / adverb
Berlin war lange Zeit eine geteilte…<br><br>*For a long time, Berlin was a divided…*	Stadt<br>*city*<br>(90%)	taxonomy	high	6/7	7	11	219,4	noun
Man soll sich morgens und abends die Zähne…<br><br>*In the morning and in the evening, one shoud…one's teeth*	putzen<br>*brush*<br>(95%)	objective relation	high	4/7	9	11	237,4	verb
Der Magier berührt den Hut mit dem…<br><br>*The magician touched the hat with the…*	Zauberstab<br>*wand*<br>(75%)	instrumental relation	medium	3/7	8	10	215,3	noun
*Der Lehrer schrieb das lange Wort an die…<br><br>*The teacher wrote the long word on the…*	Tafel<br>*blackboard*<br>(95%)	objective relation	-	4/7	9	10	220,5	noun
*Die Frau überrascht ihren…<br><br>*The wife surprises her…*	ihren Mann<br>*her husband*<br>(100%)	objective relation	-	6/7	5	5	168,6	noun
Der Chef arbeitet zu…<br><br>*The boss works too…*	lang / hause)<br>*long / home (both possible in German)*<br>(25% each)	qualitative relation	low	6/7	5	6	180	adjective / adverb
Mein neues Auto ist zuverlässig und…<br><br>*My new car is reliable and…*	schnell<br>*fast*<br>(30%)	predicative relation	low	5/7	7	11	244,9	adjective / adverb
*Das neue Buch des Autors ist…<br><br>*The author's new book is…*	gut<br>good<br>(32%)	qualitative relation	-	4/7	7	8	213,8	adjective / adverb
Das schlechte Wetter macht die Leute…<br><br>*The bad weather makes people…*	müde / traurig<br>*tired / sad*<br>(20% each)	qualitative relation	low	5/7	7	9	198,2	adjective / adverb
Der Lehrer findet den Streich gar nicht…<br><br>*The teacher doesn't think the prank is…*	lustig<br>*funny*<br>(65%)	qualitative relation	medium	4/7	8	9	228,7	adjective / adverb
*Jeden Morgen beim Frühstück las der Direktor die…<br><br>*Everyday at breakfast, the director read the…*	Zeitung<br>*newspaper*<br>(77%)	objective relation	-	5/7	9	13	270,5	noun

Der Beamte beantwortet die Frage äußerst… <br>*The official answered the question extremely…*	ernst / genau *sternly / precisely* (15% each)	qualitative relation	low	5/7	7	13	293,2	adjective / adverb
Der Bauer mäht das Gras mit der… <br>*The farmer cut the grass with the…*	Sense *scythe* (70%)	instrumental relation	medium	3/7	8	8	186,6	noun
Die Freunde konnten nicht… <br>*The friends were not able to…*	kommen / sich treffen *come / meet* (20% each)	predicative relation	low	3/7	5	5	157,6	verb
Die Feuerwehr löscht den Brand mit… <br>*The fire department extinguished the fire with…*	Wasser *water* (40%)	instrumental relation	low	5/7	8	8	192,7	noun
Die Mutter entfernt den Holzsplitter mit der… <br>*The mother removed the wood splinter with…*	Pinzette *tweezers* (85%)	instrumental relation	high	3/7	8	11	237,4	noun
Die Dame öffnet die Tür mit dem… <br>*The lady opens the door with the…*	Schlüssel *key* (40%)	instrumental relation	low	4/7	8	9	208,8	noun
Der Vertrag hat viele umstrittene… <br>*The contract has many controversial…*	Klauseln / Paragraphen *clauses / paragraphes* (25% each)	meronymy	low	4/7	6	10	206,3	noun
Unser Planet hat nur einen… <br>*Our planet only has one…*	Mond *moon* (75%)	meronymy	medium	4/7	6	8	173,2	noun
Er möchte ihr eine Tasse Tee… <br>*He wants to… her a cup of tea*	anbieten *offer* (30%)	objective relation	low	5/7	7	9	170,1	verb
Der Sportler läuft das Rennen erstaunlich… <br>*The athlete ran the race astonishingly…*	schnell *fast* (75%)	qualitative relation	medium	5/7	7	10	249,6	adjective / adverb
Pappeln sind hohe, schlanke… <br>*Poplars are high, slim…*	Bäume *trees* (90%)	taxonomy	high	5/7	5	7	187,2	noun
Der Schüler schneidet das Papier mit der… <br>*The students cuts the paper with…*	Schere *scissors* (95%)	instrumental relation	high	4/7	8	10	243,2	noun
Viele Leute finden den neuen Film…	gut *good* (35)	qualitative relation	low	6/7	7	8	225,8	adjective / adverb

Many people thought the new movie was…								
Der Bauarbeiter will die Steine…<br><br>*The construction worker wants to … the stones*	(auf)stapeln<br>*pile*<br>(20%)	objective relation	low	4/7	6	9	189	verb
Delphine hält man für besonders kluge…<br><br>*Dolphins are thought to be particularly smart…*	Tiere<br>*animals*<br>(60%)	taxonomy	medium	5/7	7	11	244,9	noun

* These sentences were not tested in the pilot study. They were later added to the stimuli set to equalize the level of constraint for both sessions. To ensure that this table contains enough information about the constraint of the sentences, we added results from our experiment. The information on the frequency of the most probable word then refers to the result from our experiment. It has to be considered that the marked items include data acquired from patients with language disorders and therefore may not fully match the healthy group of the pilot study.
