# Supplementary material for: Sentence completion in progressive supranuclear palsy following transcranial direct current stimulation
Source: NPJ Parkinsons Dis. 2023 Dec 9;9:162. doi: 10.1038/s41531-023-00610-0 (PMC10710437; doi:10.1038/s41531-023-00610-0)
Supplement: Supplementary file 2 — Related Manuscript File [file 41531_2023_610_MOESM2_ESM.pdf]

nature portfolio

Corresponding author(s): Johanna Scholtz
Last updated by author(s): Sep 24, 2023

Reporting Summary

Nature Portfolio wishes to improve the reproducibility of the work that we publish. This form provides structure for consistency and transparency in reporting. For further information on Nature Portfolio policies, see our Editorial Policies and the Editorial Policy Checklist.

Please do not complete any field with "not applicable" or n/a. Refer to the help text for what text to use if an item is not relevant to your study.
For final submission: please carefully check your responses for accuracy; you will not be able to make changes later.

Statistics

For all statistical analyses, confirm that the following items are present in the figure legend, table legend, main text, or Methods section.

n/a	Confirmed	
☐	☒	The exact sample size (*n*) for each experimental group/condition, given as a discrete number and unit of measurement
☐	☒	A statement on whether measurements were taken from distinct samples or whether the same sample was measured repeatedly
☐	☒	The statistical test(s) used AND whether they are one- or two-sided<br>*Only common tests should be described solely by name; describe more complex techniques in the Methods section.*
☐	☒	A description of all covariates tested
☐	☒	A description of any assumptions or corrections, such as tests of normality and adjustment for multiple comparisons
☐	☒	A full description of the statistical parameters including central tendency (e.g. means) or other basic estimates (e.g. regression coefficient) AND variation (e.g. standard deviation) or associated estimates of uncertainty (e.g. confidence intervals)
☐	☒	For null hypothesis testing, the test statistic (e.g. *F*, *t*, *r*) with confidence intervals, effect sizes, degrees of freedom and *P* value noted<br>*Give P values as exact values whenever suitable.*
☒	☐	For Bayesian analysis, information on the choice of priors and Markov chain Monte Carlo settings
☒	☐	For hierarchical and complex designs, identification of the appropriate level for tests and full reporting of outcomes
☒	☐	Estimates of effect sizes (e.g. Cohen's *d*, Pearson's *r*), indicating how they were calculated

Our web collection on statistics for biologists contains articles on many of the points above.

Software and code

Policy information about availability of computer code

Data collection	Data was collected using Microsoft Excel
Data analysis	Jamovi software (2.0.0.0)

For manuscripts utilizing custom algorithms or software that are central to the research but not yet described in published literature, software must be made available to editors and reviewers. We strongly encourage code deposition in a community repository (e.g. GitHub). See the Nature Portfolio guidelines for submitting code & software for further information.

Data

Policy information about availability of data

All manuscripts must include a data availability statement. This statement should provide the following information, where applicable:
- Accession codes, unique identifiers, or web links for publicly available datasets
- A description of any restrictions on data availability
- For clinical datasets or third party data, please ensure that the statement adheres to our policy

The datasets used and/or analysed during the current study are available from the corresponding author on reasonable request.

Research involving human participants, their data, or biological material

Policy information about studies with human participants or human data. See also policy information about sex, gender (identity/presentation), and sexual orientation and race, ethnicity and racism.

Reporting on sex and gender	The term „sex“ was used, since biological attribute was considered in this study
Reporting on race, ethnicity, or other socially relevant groupings	N/A
Population characteristics	5 PSP patients (age 72.6 ± 5.8; duration of illness 3.6 ± 1.7), 4 MSA patients (age 55.8 ± 4.1; duration of illness 4.5 ± 1.7), 14 healthy elderlies (age 64.5 ± 13.3)
Recruitment	PSP & MSA group were recruited by Hospital Lippe-Lemgo & support groups. HE recruited by University Bielefeld database.
Ethics oversight	The study was approved by the Ethics Committee of Bielefeld University, Germany (approval number 2021-072)

Note that full information on the approval of the study protocol must also be provided in the manuscript.

Field-specific reporting

Please select the one below that is the best fit for your research. If you are not sure, read the appropriate sections before making your selection.

[X] Life sciences [ ] Behavioural & social sciences [ ] Ecological, evolutionary & environmental sciences

For a reference copy of the document with all sections, see nature.com/documents/nr-reporting-summary-flat.pdf

Life sciences study design

All studies must disclose on these points even when the disclosure is negative.

Sample size	5 PSP, 4 MSA, 14 healthy elderlies (23 in total)
Data exclusions	No data were excluded from quantitative analyses
Replication	There was no attempt to replicate the findings
Randomization	Randomization of order of stimulation (tDCS/sham or sham/tDCS); Randomization of tasks in the sessions, Randomization in task design
Blinding	Triple-blinded design: Subjects & experimenter were blind for stimulation (tDCS/sham) in the sessions and analysis of language data

Behavioural & social sciences study design

All studies must disclose on these points even when the disclosure is negative.

Study description	
Research sample	
Sampling strategy	
Data collection	
Timing	
Data exclusions	
Non-participation	
Randomization	

Ecological, evolutionary & environmental sciences study design

All studies must disclose on these points even when the disclosure is negative.

Study description	
Research sample	
Sampling strategy	
Data collection	
Timing and spatial scale	
Data exclusions	
Reproducibility	
Randomization	
Blinding	

Did the study involve field work? ☐ Yes ☐ No

Field work, collection and transport

Field conditions	
Location	
Access & import/export	
Disturbance	

Reporting for specific materials, systems and methods

We require information from authors about some types of materials, experimental systems and methods used in many studies. Here, indicate whether each material, system or method listed is relevant to your study. If you are not sure if a list item applies to your research, read the appropriate section before selecting a response.

Materials & experimental systems

n/a	Involved in the study	
x	☐	Antibodies
x	☐	Eukaryotic cell lines
x	☐	Palaeontology and archaeology
x	☐	Animals and other organisms
x	☐	Clinical data
x	☐	Dual use research of concern
x	☐	Plants

Methods

n/a	Involved in the study	
x	☐	ChIP-seq
x	☐	Flow cytometry
x	☐	MRI-based neuroimaging

Antibodies

Antibodies used	n/A
Validation	n/A
